# PERMANENT TRANSITION OF HOMECARE RECIPIENTS WITH DEMENTIA TO NURSING HOMES IN NYS: RISK FACTORS

**DOI:** 10.1093/geroni/igz038.1526

**Published:** 2019-11-08

**Authors:** Yuchi Young, Maksim Papenkov, Wan-Hsaing Hsu, Faryal Shahid, Yen-Hong Kuo

**Affiliations:** 1 SUNY at Albany, Albany, New York, United States; 2 New York State Department of Health, Albany, New York, United States; 3 Hackensack Meridian Health, Neptune, New Jersey, United States

## Abstract

Objective. To determine predictors associated with the permanent transition to nursing homes among home care recipients with dementia. Methods. Retrospective cohort study (01/2007-12/2012). Study participants (n=48,338) include older adults age 65+ with dementia who received home health services in NYS for at least 2 months prior to permanent transition to nursing homes. Permanent transition is defined as discharge from a home care agency to a nursing home where participants must reside for 3+ months continuously. Two data sets were used—the Minimum Data Set (MDS) and Outcome and Assessment Information Set (OASIS). Multivariate logistic regression was used to quantify the association between predictors and permanent transition to nursing homes. Results: 29% of home care recipients with dementia made permanent transitions to nursing homes. The mean age was 83 years old ranging from 65-111. Among the permanent transition group, 47% were age 85+, 68% female, 75% white, 13% black, 9% Hispanic, 29% with both urinary & bowel incontinence, and 15% with depression. Risk factors associated with permanent transition included increasing age (OR=1.1; 95% CI 1.03-1.18); being white (OR =1.25; 95%CI 0.83-0.94) compared to black, urinary and bowel incontinence vs. continence (OR=1.46; 95% CI 1.37-1.56); depression vs. no depression (OR=1.2; 95% CI 1.11-1.25); hip fracture vs. no hip fracture (OR=2.63; 95% CI 2.27-3.05), and 3+ hospitalizations vs. no hospitalization (OR=3.02; 95% CI 2.77-3.29). Conclusion: Two potential modifiable risk factors related to permanent transition to nursing homes are depression and incontinence. Early diagnosis and treatment may delay or avert nursing home entry.

